# The need for stronger international support to integrate health and well-being and transform education: a perspective on developing countries

**DOI:** 10.3389/fpubh.2024.1415992

**Published:** 2024-09-05

**Authors:** Parviz Abduvahobov, Stuart J. Cameron, Ayodeji Ibraheem, Joanna Herat, Christopher Castle

**Affiliations:** Health and Education Section, Division for Peace and Sustainable Development, Education Sector, UNESCO, Paris, France

**Keywords:** education, school, cross-sectoral planning, school health, school nutrition, well-being, child health, adolescents

## Abstract

The reciprocal relationship between education and health is well-established, emphasizing the need for integrating health, nutrition, and well-being components into educational sector planning. Despite widespread acknowledgment of this need, countries lack concrete measures to achieve this integration. We examine challenges that countries have faced and the progress they have made in integrating these components into education sector plans and review the extent to which existing educational planning guidelines and tools address health and well-being. The review reveals a significant underrepresentation of health, well-being, and related themes in existing educational planning frameworks. Recent tools and frameworks developed to support a more holistic approach to education have not yet been widely adopted in standard education sector planning processes. The implementation of such approaches remains inconsistent, with significant barriers including limited cross-sectoral collaboration, lack of capacity, and insufficient funding, among others. Addressing these gaps requires improved guidance, technical support, and a multisectoral approach to education planning that includes health, nutrition, and well-being as fundamental components of foundational learning, supported by political commitment, capacity, and adequate financing.

## Introduction

The nexus between education and health has been extensively studied and firmly established (1). Children and adolescents who are healthy, well-nourished, engage in regular physical activity, and study in a safe environment learn better (2–5). Conversely, educational attainment has a predominantly beneficial effect on a broad spectrum of health outcomes (6). Recognizing this reciprocal relationship, it is now rare to encounter a country that has not adopted school health and nutrition (SHN) policies or provided health services in school settings at some level (7). The unprecedented school closures triggered by the COVID-19 pandemic have not only clarified the connections between health, well-being, and learning but have also emphasized the critical role of schools in ensuring educational continuity as well as in supporting physical health and addressing emotional and mental health challenges.

Building upon this understanding, the 2022 Transforming Education Summit (TES) has brought to light the global commitment to addressing these challenges and transforming education. Analysis of 143 national statements of commitment post-TES reveals a widespread acknowledgment of the need to support students’ and teachers’ psycho-social and mental well-being, with nearly 60% of countries advocating for enhanced physical and mental health and safety measures (84 out of 143 countries) (8, 9). This analysis serves as a crucial barometer for understanding global education sector priorities and commitments toward integrating health and well-being in the aftermath of the pandemic.

Despite the clear recognition of these needs, very few countries have articulated concrete measures to achieve them (8). This gap between commitment and implementation underscores the urgency for the school health community, United Nations, and development partners to provide support in actualizing this vision. Education stakeholders may see health and well-being as competing with other pressing priorities, such as the need to respond to learning poverty.^1^ A key role for UNESCO and its partners is to demonstrate how health and well-being are also fundamental aspects of learning and can be part of an approach that supports foundational learning^2^ through a holistic understanding of learners’ needs. This involves advocacy, but importantly, it also involves improving the guidance and provision of technical support on how to achieve these goals in practice.

## Challenges and progress in integrating health and well-being in education planning

Countries are increasingly considering how to transform their education systems and integrate health and well-being, albeit at varying levels, influenced by national context and priorities. Pioneering approaches in Kenya and Indonesia demonstrate the effectiveness of a unified government strategy involving multiple line ministries, including education, health, and home affairs, to promote inclusive education through comprehensive SHN programs (12). From 2000 to 2015, Sub-Saharan Africa saw a notable rise in health and nutrition interventions within education sector plans (ESPs) (13).

However, this progress has been uneven, with varying coverage and implementation. Country-specific analyses have shown diverse levels of SHN policies and interventions, often aimed at promoting equity and inclusion (14, 15). Some examples from diverse regions illustrate this variation: In Egypt, the 2023–2027 ESP recognizes the constrained resources for school feeding, water, sanitation, and hygiene (WASH) as an important barrier. Nepal’s 2022/23–2031/32 ESP includes strategies for basic health and nutrition services, WASH, and addressing abuse, discrimination, and bullying. Liberia’s 2022/23–2026/27 ESP provides for skill-based health education through girls’ clubs, nutrition services, and reducing gender-based violence. The level of ambition in relation to school health and well-being is evident, but all three plans also highlight significant financing gaps.

For many such countries attempting to address health and well-being in schools, questions remain about the feasibility of interventions reaching national coverage. Often, the evidence basis for interventions is unclear; they are relatively small in scale and are insufficiently funded. Many plans lack an overall theory of change or references to globally established definitions and frameworks (14). Cooperation between sectors and human resource capacity is often not in place to implement these plans (15). Deficiencies in strategic planning knowledge and skills and low levels of stakeholder engagement prevent these intersectoral planning processes from working as intended (16).

## Gaps in existing educational planning tools and processes

In the education sector, a number of tools have played an essential role in supporting analysis and planning. Developed primarily between 2014 and 2021, these include the Guidelines for Education Sector Plan Preparation (17) and the Education Sector Analysis methodological guidelines (18–20).

Despite their wide usage and appreciation within the global education sector, these tools do not yet provide the support that countries need when it comes to health and well-being. In particular, recent work recognizes the transformative potential of education – to empower learners to make informed decisions and take actions concerning their health, nutrition, well-being, global citizenship, and issues such as climate change and peace (21, 22). A keyword analysis reveals a stark underrepresentation of these essential themes and the need for cross-sectoral work in education sector planning tools and in guidelines such as those produced by the Global Partnership for Education (GPE), indicating a significant disconnect between current educational strategies and evolving global challenges (Table 1).

**Table 1:** Keyword search.

	Guidelines for education sector plan preparation (17)	GPE’s Education System Enabling Factor Analysis (23)	GPE application for grants (24)	GPE’s Quality standards for grant applications (25)
Gender	19	48	13	3
Health	0	3	0	0
Well-being	0	0	0	0
Climate	0	4	0	0
Green*ing	0	0	0	0

These documents primarily emphasize ‘traditional’ education metrics such as enrolment and internal efficiency, and discussions on ‘health’ are often confined to screening and health service provision (18). They do not incorporate the more recent specialized tools designed to help countries and education systems collect data, assess the status of their policies and practices, and identify areas for improvement in order to promote learners’ health, safety, and well-being. The following sections describe some of these tools, with suggestions on how they can be used more effectively.

## New tools to help bridge the gap

Since the early 2000s, many new tools have been developed, often the result of burgeoning cooperation among international organizations in this arena. Specifically, there are now multiple (i) frameworks and guidelines, (ii) policy diagnostic tools, (iii) evidence reviews, (iv) survey instruments, and (v) global data sources that can support this work.

Key *frameworks* include Focusing Resources on Effective School Health (FRESH), launched by UNESCO, UNICEF, WHO, and the World Bank during the World Education Forum, Dakar, in April 2000 (26). FRESH is an intersectoral framework for promoting educational success, health, and development of school-age children and adolescents and has expanded over time to generate a set of linked diagnostic tools for analysis of topics, such as how contexts may support or undermine school health, the status of evidence generation in each country, what resources are committed to school health, and the prioritization of specific policy areas or programs.

Under its Systems Approach for Better Education Results (SABER) initiative, the World Bank drew on FRESH to produce ‘What Matters Most for School Health and School Feeding’ (27), presenting a conceptual framework, a set of policy goals, and guidance around the trade-offs that may be involved in implementing school health and school feeding. Health-promoting schools—a long-standing whole-school approach capitalizing on the potential of schools to foster the physical, social–emotional, and psychological conditions for better health as well as educational attainment—has been advanced in recent years with the development of a set of global standards and indicators, to support governments in embedding the approach in all aspects of education systems (28).

Such frameworks are needed in education sector planning to ensure that the analysis of health in education, dialog and consultation around it, and the planned policy and program responses are holistic and coherent, reflecting a suitable level of ambition in terms of education and health interventions that mutually support each other. In addition, standards and indicators have recently been developed around specific areas, such as sexuality education (29, 30) and safety from violence in and around schools (31). Frameworks for ending violence against children include INSPIRE and Safe to Learn (32, 33), and the INSPIRE toolkit includes specific and extensive guidance on implementation, adaptation, scale-up, and results indicators (34–36). These help planners design concrete and consistent sets of activities.

*Policy diagnostic tools* guide policymakers and planners through analyzing the components of school health and well-being to reach a better understanding of what the barriers are, the strengths and weaknesses of current policies and programs, and what adaptations and new resources might be needed to make faster progress. These are particularly important for education sector analysis at the start of a planning cycle. Such tools are often linked to the frameworks listed above, as is the case for those developed as part of SABER, FRESH, Safe to Learn (37), and the Sexuality Education Review and Assessment Tool (SERAT) (38). UNICEF (39) developed a checklist tool for promoting effective and equitable learning recovery following COVID-19 school shut-downs, which includes consideration of services to meet children’s learning, health, nutrition, psycho-social well-being, and other needs through cross-sectoral collaboration and to ensure protection, safety and referral systems.

In many countries, education reforms focused on health and nutrition will involve new areas of programming, and countries will need to review practices that have been effective globally and adapt these to local contexts. A number of global *evidence reviews* now exist to support this process, consolidating the best available global evidence on interventions in relation to school health and well-being. Systematic reviews of interventions to improve access to and learning in school have analyzed the impacts of school-based health and nutrition programs (40). A volume in the Disease Control Priorities series presented the latest evidence on high-return investments in school health and proposed a package of interventions for countries to consider implementing (41). The INSPIRE guidelines developed by WHO, UNICEF, and others (32) propose seven strategies for ending violence against children and document the evidence and country cases for each strategy.

*Survey instruments* have been developed that improve our understanding of the health and well-being of school-age children and adolescents and the extent to which their home and school environments promote better health. Where these have already been carried out, they can inform education sector analysis, but many countries may need to enhance their existing routine data collection as part of their education management information systems (EMIS), and sometimes as monitoring and evaluation mechanisms for new programs.

Major international household survey initiatives, such as Demographic and Health Surveys (DHS), UNICEF’s Multiple Indicator Cluster Surveys (MICS), and the World Bank-supported Living Standards Measurement Surveys (LSMS), have long collected data on health, immunization, water, sanitation, hygiene, food security, stunting, and early childhood development; and relatively recent additions cover aspects such as child discipline and disability in standardized formats. The Global School-Based Student Health Survey (42) measures and assesses the behavioral risk factors and protective factors related to the leading causes of morbidity and mortality among students, while the Violence Against Children and Youth Survey (43) measures physical, emotional, and sexual violence against children and youth up to age 24, both in and outside of school.

Facility-based surveys include the Global School Health Policies and Practices Survey (44), which asks head teachers about health services, the physical environment, food and nutrition, health education, physical education, governance and leadership, and school policies and resources. The Brief Early Childhood Quality Inventory (45)—a checklist for analyzing the quality of early childhood development centers—also incorporates items on clean and safe environments and physical punishment. While most countries have conducted at least some of these surveys, low and middle-income countries continue to struggle with insufficient recent data for analysis and planning.

*Global data sources* enable countries and international partners to rapidly access the results from these surveys and compare them across countries. These are important tools for national education sector analysis, allowing countries to understand how they stand in relation to comparator countries, and helping global actors shape their support.

The UNESCO Institute for Statistics (UIS) tracks the indicators for Sustainable Development Goal 4, including the number of young children who are developmentally on track in health, learning and psycho-social development; the extent to which global citizenship education and education for sustainable development are mainstreamed in national education policies; the percentage of schools providing life skills-based HIV and sexuality education; percentage of students showing understanding of global citizenship and sustainability; the percentage of students showing proficiency in environmental science or geoscience; proportion of schools with access to water, sanitation and hygiene facilities; bullying; and, attacks on students, personnel and institutions. However, the availability of data on these indicators remains limited in many cases. The annual *Global Education Monitoring Report* tracks progress on the full range of education indicators around the world, while *Ready to Learn and Thrive* (7) summarizes the data that are available on SHN and highlights the many gaps that remain.

UNICEF’s Foundational Learning Action Tracker tracks the number of countries implementing nationwide policy measures to improve foundational learning, including the development of psycho-social support and well-being (46). The school violence dataset developed by the Center for Global Development (47) brings together DHS, VACS, and several other sources to document the extent and nature of school violence around the world. The creators of this dataset highlight that, despite an increasingly large number of surveys collecting information on violence, most counties still lack actionable data to address school-related violence.

Together, these tools and resources represent a growing body of work that can support national policymakers and their international partners in incorporating health and well-being into their education sector planning. The resources highlight many of the gaps in data and evidence that remain. However, filling these gaps should in itself be part of the cross-sectoral planning and evidence-informed policymaking processes, and many of the instruments and frameworks needed to do so already exist.

## Discussion

The frameworks, guidelines, tools, evidence reviews, diagnostics, and data sources listed above, most of which are relatively recent, fill important gaps in the ability of countries to improve health and well-being in schools. The tools can be brought in at each stage in the education sector planning cycle (17):

- *Education sector analysis* can increasingly draw on data from new surveys, diagnostic tools, and global sources.
- *Prioritization and consultation exercises* can use health and education frameworks to ensure a holistic dialog, reflecting the potential for mutual benefit across the two sectors and help delineate responsibilities between different actors in the sectors.
- *Program design and implementation planning* can use the same frameworks to ensure a coherent and comprehensive response, both for the sector as a whole and for specific areas of intervention, such as WASH or sexuality education, and can draw on new evidence reviews to form appropriate responses to health and education challenges.
- *Monitoring and evaluation* of education programs can use the new frameworks and evidence reviews to develop theories of change and can use the new survey instruments and diagnostic tools to develop indicators and standards to measure progress.

However, these tools have not yet been integrated into the standard tools and processes of international support to education sector planning and may be difficult for education planners to put into practice. To address this, it is essential to develop a clear implementation strategy, which includes a commitment by the UN and development partners to create a comprehensive handbook that consolidates all frameworks, tools, and evidence reviews. This handbook should be made available to education sector planners in countries and promoted by development partners, UN organizations, and GPE. Building local capacity within the relevant departments of the ministries is imperative, ensuring education sector planners are trained on the use of these tools. Additionally, establishing a support network for cross-country learning and technical support, where countries can share experiences and best practices, is crucial.

The tools vary in their scope from the specific to the holistic, but it is essential that they be seen as part of a wider, holistic vision of transformative education—education that supports learners’ health, well-being, and understanding of sustainable development, peace, and citizenship. Such a vision provides a framing that can bring together these diverse elements and help education planners build them into a cohesive strategy. Moreover, it is essential for the wider vision to articulate how health and well-being are essential to foundational learning and ending ‘learning poverty’. The recent Commitment to Action on Foundational Learning, endorsed by many governments and international organizations, does this, recognizing the need to support the health, nutrition, and psycho-social well-being of every teacher and child and, in turn, recognizing how foundational learning contributes to productive citizenship, sustainable development, gender equality and other national goals (10, 11, 48).

A core aspect of this transformative vision is for education sector planning and policymaking to become more multisectoral, drawing in health, community development, and other social sectors to support child development. The need for deeper cross-sectoral coordination and engagement of other sectors in education sector planning has long been recognized, particularly for early childhood development programming (49), yet remains an aspiration in many cases.

Regardless of the availability of frameworks and tools, their impact will be limited if stakeholders, particularly donors and development partners, do not actively utilize them in their support to countries. There is a risk that external assistance remains driven by each donor’s priorities rather than the needs identified in the evidence generated within each country. This could potentially hinder the effective implementation of a comprehensive school health agenda, deviating from a truly learner-centered model.

The development of a better and more integrated set of tools does not in itself guarantee better policy or practices on health and well-being in schools. Many governments, in particular the education authorities, will need direct technical support or training to make the best use of these tools, backed up by mechanisms for cross-country support and learning, and a coherent framework is needed to guide the different international bodies offering this support. The integration of evidence into policymaking and planning involves a complex process in which political, ideological, and economic factors all play a role (50, 51). A supportive political economy and domestic financing, in particular, will be necessary in the longer term to sustain financing for school health programs. Showing that there is already a strong global evidence base for such programs and coherent, integrated technical guidance on how they can be put into practice is an essential foundation for such political and financial resources to be mobilized.

## Data Availability

The original contributions presented in the study are included in the article/supplementary material, further inquiries can be directed to the corresponding author.

## Footnotes

^1^Learning poverty means being unable to read and understand a simple text by age 10 (10).

^2^Foundational learning refers to basic literacy, numeracy, and transferable skills such as socio-emotional skills (11).
